# Artificial Intelligence Approaches to Modeling Equivalent Circulating Density for Improved Drilling Mud Management

**DOI:** 10.1021/acsomega.5c02050

**Published:** 2025-04-28

**Authors:** Mohammad-Saber Dabiri, Reza Haji-Hashemi, Abdolhossein Hemmati-Sarapardeh, Reza Zabihi, Mohammad-Reza Mohammadi, Mahin Schaffie, Mehdi Ostadhassan

**Affiliations:** † Department of Petroleum Engineering, Shahid Bahonar University of Kerman, Kerman 76169-141111, Iran; ‡ State Key Laboratory of Petroleum Resources and Prospecting, China University of Petroleum (Beijing), Beijing 102249, China; § State Key Laboratory of Continental Shale Oil, Northeast Petroleum University, Daqing, 163318, China; ∥ Institute of Geosciences, Marine and Land Geomechanics and Geotectonics, Christian-Albrechts-Universität, Kiel 24118, Germany

## Abstract

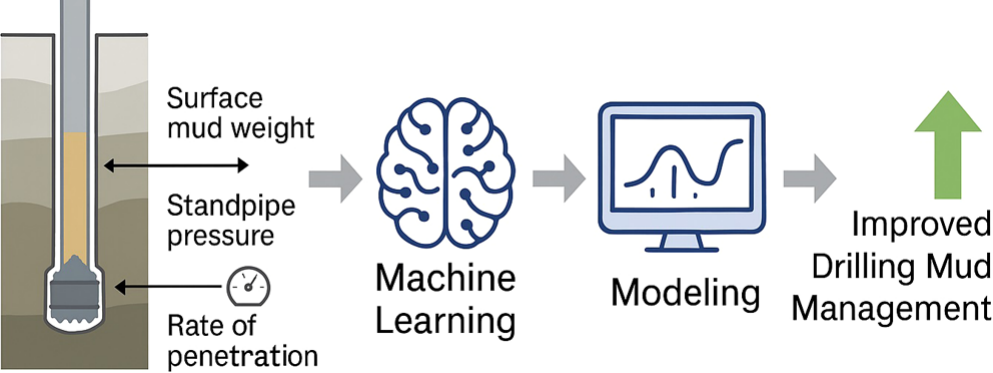

Proper management of equivalent circulating density (ECD) plays a crucial role in drilling processes since poor control can lead to serious well control problems such as lost circulation and formation fracturing. Traditionally, ECD has been calculated using downhole tools or mathematical models. The novelty of this study lies in the use of fewer inputs for modeling, which enhances the simplicity and efficiency of the approach. Furthermore, several advanced machine learning models have been employed to predict ECD under standard conditions. The models utilized in this study demonstrated high adaptability to complex data and varying conditions, resulting in superior performance in predicting ECD compared to other models. Additionally, these models exhibited robustness to noisy and inconsistent data, enabling accurate predictions even in the presence of discontinuous and irregular data sets. Moreover, the empirical relationship developed in this study outperforms existing relationships in terms of accuracy, offering a more reliable and trustworthy predictive framework. To this goal, a data set containing 2367 field measurements from two wells in an Iranian oilfield using water-based fluids (WBF) was employed. Of these data, 70% was utilized for model development and training, while 30% was reserved for testing. Key variables influencing ECD, including standpipe pressure (SPP), rate of penetration (ROP), and surface mud weight (MW), were analyzed. Seven advanced machine learning algorithms were applied: cascade forward neural network (CFNN), generalized regression neural network (GRNN), wavelet neural network (WNN), and support vector regression (SVR) optimized with particle swarm optimization (PSO-SVR), farmland fertility algorithm (FFA-SVR), and grasshopper optimization algorithm (GOA-SVR). Additionally, a mathematical correlation was developed using the group method of data handling (GMDH). The results indicated that while all models accurately predicted ECD, the GOA-SVR algorithm provided the most reliable outcomes, with average absolute percent relative errors (AAPRE) values of 0.0823, 0.0975 and 0.0869% for the training, testing, and entire data sets, respectively. Moreover, the GMDH model demonstrated superior performance compared to other existing empirical models, especially when three key input variables were utilized. Additionally, the sensitivity analysis revealed that the surface mud weight had the most significant influence on ECD prediction. Finally, the leverage technique was implemented to assess the operational scope of the GOA-SVR and GMDH models. For the GOA-SVR model, 59 data points (∼2.5%) were identified as suspicious, while for the GMDH model, 29 data points (∼1.3%) were flagged. Additionally, 30 data points (∼1.3%) for GOA-SVR and 42 data points (∼1.8%) for GMDH were recognized as potential outliers, indicating that despite accurate predictions, these points fall outside the models’ applicability domain.

## 1 Introduction

Drilling fluid, which accounts for approximately 20% of operational costs, plays a crucial role in drilling activities and has been extensively studied in reservoir engineering.^1^ Optimizing and controlling drilling fluid parameters helps reduce risks like wellbore instability, pipe sticking, and fluid loss while preventing potential blowouts and the abandonment of wells.^2−5^ Inadequate management of equivalent circulating density (ECD), which is recognized as a fundamental parameter in drilling engineering and well control, can result in lost circulation, a phenomenon that incurs significant economic challenges.^6,7^ When the ECD surpasses the formation pore pressure, the drilling mud penetrates the formation, resulting in lost circulation. Conversely, if the ECD falls below the formation pore pressure, the risk of gas kick increases. Thus, an in-depth understanding and accurate estimation of bottom-hole ECD are crucial for efficient formation pressure control and optimizing drilling performance.^8−10^ Multiple variables influence the ECD during drilling operations, such as standpipe pressure (SPP), concentration of cuttings, drill pipe rotation, mud density, as well as downhole temperature and pressure. In the literature, two primary methods are commonly used to estimate ECD. The first method involves the use of pressure while drilling (PWD) and measurement while drilling (MWD) systems for continuous monitoring of bottom-hole pressure during the drilling process.^11^ These systems are equipped with high-precision quartz sensors capable of accurately measuring ECD, annular pressure, and internal pressure. This method is regarded as the most dependable, particularly in challenging well conditions and high-risk environments. However, despite its high accuracy, it is costly and cannot provide predictions for undrilled sections of the well. The second method for calculating ECD is the use of a mathematical model, which is calculated as follows:
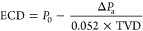
1This formula calculates the ECD using three key parameters. *P*_0_ represents the hydrostatic pressure, which is the pressure exerted by the mud column at the surface. Here, Δ*P*_a_ denotes the annular pressure loss, which is the pressure drop in the annular space due to flow resistance. Lastly, TVD stands for true vertical depth, which is the vertical distance from the surface to the point of interest in the wellbore. The annular friction pressure loss is the sole unknown variable, while all other variables are predetermined. Thus, accurately calculating the annular pressure is essential for ensuring operational reliability and well control. The value of annular pressure loss is influenced by various factors, including drilling fluid properties, pump flow rate, annular geometry, wellbore trajectory, gas intrusion, and additional parameters. Accurate calculation of annular pressure loss typically involves assuming a concentric and circular borehole cross-section. As a result, hydraulic simulation software was created to compute the bottom-hole ECD and generate advanced forecasts for the undrilled intervals of the well. Different rheological models, including power law, Bingham plastic, and Herschel-Bulkley, were employed to foretell ECD and SPP. However, it was observed that each rheological model requires distinct input parameters, and discrepancies were observed between the predicted ECD and the values measured by PWD.^12,13^

### 1.1 Literature Review

Machine learning (ML) techniques have been widely used as advanced methods in various sciences in recent years.^14−18^ These approaches enable the processing and analysis of large-scale drilling data, identifying intricate patterns that traditional methods may overlook, and utilizing these insights to enhance predictive accuracy and optimize decision-making processes. Given the inherent limitations and inconsistencies in hydraulic models and mathematical correlations, researchers have increasingly explored ML-based approaches as a cost-effective alternative for ECD prediction, leveraging surface drilling data. The subsequent section provides an overview of key studies in this domain.^19^

Elzenary et al.^20^ applied a multilayer perceptron (MLP) neural network model and an ANFIS to estimate the ECD. They introduced MW_S_, ROP, and the pressure in the drilling string as input variables. Their results showed that both created networks have a good ability to predict drilling fluid density. Rahmati and Tatar^21^ created a radial basis function (RBF) algorithm using a database of 884 oil-based fluid (OBF), water-based fluid (WBF), synthetic oil fluids, and electronic fluids. The model’s input parameters including fluid type, surface density, fluid temperature, and pressure were selected. The results indicated that the RBF has higher accuracy than other previous networks. Alkinani et al.^22^ utilized an artificial neural network (ANN) model consisting of one hidden layer having 12 neurons to forecast ECD. The study used data from 2000 wells, incorporating key parameters such as the mud inversion point, plastic viscosity, string rotation speed, pump flow rate, weight on the bit, and bit nozzle surface area, to predict the ECD. Their results indicated that the perceptron network, utilizing the Bayesian Regularization (BR) training algorithm, outperformed other methods. Abdelgawad et al.^23^ developed an ECD forecasting model that integrates ANN and ANFIS. The ANFIS model employed Gaussian membership functions (GMF) for the inputs and linear membership functions for the outputs. Their ANN model was designed with a hidden layer comprising 20 neurons. The findings showed that the neural network with an APRE of 0.2264% and a R^2^ of 0.99 provides the best results. Ahad et al.^24^ investigated intelligent models including SVR models and fuzzy logic to prognosticate the features of rock and drilling fluid. The findings showed that the error backpropagation model with gradient descent training algorithm (BP-GDL) with an ARE of 0.367% and an *R*^2^ of 0.999 provides the best results. Al-Rubaii et al.^25^ designed a resilient model incorporating artificial intelligence (AI) approaches to achieve highly accurate estimates of drilling fluid density and the ECD. Their findings demonstrated that ANNs and support vector machines (SVM) could anticipate the ECD with an *R*^2^ of 0.9947 and an AAPRE% of just 0.23. The decision tree (DT) model also achieved mud weight estimation with an *R*^2^ of 0.9353 and an AAPRE% of 1.66%. Ekechukwu et al.^26^ employed the XGBoost model to predict the ECD in drilling operations. The model showed excellent accuracy, yielding an *R*^2^ value of 1 and an RMSE of 0.0005% on training data, and an *R*^2^ of 0.989 with an RMSE of 0.023% on testing data. Additionally, relevancy factor analysis revealed that mud weight (MW), weight on hook (WOH), and SPP were the most influential factors in ECD predictions. Lastly, Dabiri et al.^27^ applied advanced ML techniques to forecast mud density under HPHT conditions. They employed 986 experimental data points for modeling this parameter. Based on their findings, the most accurate predictions of mud weight were achieved by the PSO-SVR model excluding solid content as an input, and the WNN model incorporating it, with AAPRE values of 0.5569 and 0.2475%, respectively. Table 1 summarizes additional research on the application of AI for predicting ECD.

**Table 1:** Several Studies on the Application of ANNs to Estimate ECD

references	model	input parameter	model performance
Ahmadi et al.^28^	PSO-ANN, FIS, GA-FIS	pressure, temperature, initial density	*R*^2^ = 0.9964
			AAPRE = 0.0001374
Alkinani et al.^29^	ANN	flow rate, weight on bit, TFA, mud weight, yield point, plastic viscosity, RPM	*R*^2^ = 0.999
Alsaihati et al.^19^	SVM-RF	flow rate, standpipe pressure, hook load, torque, drill string speed, weight on bit, rate of penetration,	*R*^2^ = 0.95–0.99
			RMSE = 0.23 to 0.42
Gamal et al.^30^	ANN and ANFISs	penetration rate, weight on bit, rotating speed, torque, pumping rate, and pressure of standpipe	*R*^2^ = 0.98
			AAPE = 0.3
Al-Rubaii et al.^25^	ANN and SVM	pump flow rate, yield point, initial mud weight, plastic viscosity, standpipe pressure, rate of penetration, low shear yield point	*R*^2^ = 0.9947
			AAPE = 0.23
Ekechukwu et al.^31^	XGBoost	rate of penetration, weight on the hook, surface torque, mud weight in, mud weight out, weight on the bit, standpipe pressure, total gas out, pump flow, surface RPM.	*R*^2^ = 0.989
			RMSE = 0.23

### 1.2 Purpose and Novelty of This Study

As previously mentioned, mathematical models often lack precision, and downhole measurements are both costly and subject to operational constraints. Consequently, achieving high accuracy in determining the ECD is essential.^32^ This study seeks to address this need by applying AI models with the highest possible accuracy. Unlike earlier studies that used a greater number of parameters, our model utilizes fewer parameters, offering several advantages: reduced computational complexity, faster processing times, and a lower risk of overfitting, all while preserving accuracy. Additionally, a mathematical relationship was developed that outperforms previously developed equations in terms of accuracy. Moreover, advanced ML techniques was implemented that, to the best of our knowledge, have not been previously applied in this context.

This study leverages a comprehensive data set of 2367 data points from oil wells to develop advanced intelligent techniques for predicting the ECD. The work employs multiple robust AI models, including a cascade forward neural network (CFNN) trained using Levenberg–Marquardt (LM) and Bayesian regression (BR) algorithms. Additionally, predictive techniques such as the wavelet neural network (WNN), general regression neural network (GRNN), support vector regression (SVR) combined with the farmland fertility algorithm (FFA-SVR), particle swarm optimization (PSO-SVR), and the grasshopper optimization algorithm (GOA-SVR) are applied. The research also investigates mathematical relationships through the group method of data handling (GMDH) and performs a sensitivity analysis to assess the impact of input variables. Ultimately, the leverage approach is implemented to verify the precision of real-field data and assess the dependability of best-developed models for ECD estimation.

## 2 Data Gathering

In this research, an extensive data set was compiled to anticipate the ECD. A total of 2367 real WBF data points were collected from Iranian oilfields, with SPP, ROP, and MW serving as the input parameters for the modeling process. The supplied data was divided into two categories before the development of the models. The training and testing subsets represent 70 and 30% of the entire data set. The test data evaluated the precision with which the developed system predicts results based on new input variables, while the training data was applied to train the model. The statistical information regarding the ECD databank employed for modeling is presented in Table 2. The broad range of ROP (0.27–18.60 m/h), MW (1.03–1.70 g/cm^3^), and SPP (2.72–26.92 MPa) may result in a robust, overarching model for predicting ECD.

**Table 2:** Statistical Analysis of Data Applied in This Work

mud type	ROP (m/h)	MW (g/cm^3^)	SPP (MPa)	ECD (g/cm^3^)
**water-based**				
average	5.12	1.21	13.82	1.22
median	4.83	1.19	13.44	1.20
mode	5.00	1.11	12.29	1.12
kurtosis	1.30	4.06	0.23	3.93
skewness	0.65	2.12	0.09	2.10
minimum	0.27	1.03	2.72	1.04
maximum	18.60	1.70	26.92	1.71

## 3 Methodology

This research adopts a comprehensive methodological approach to explore the role of AI in scientific research processes, with a particular emphasis on assessing ECD through AI-based models. To enhance understanding and facilitate reproducibility, the model creation procedure was organized into the following stages:

- Data collection:

1. Database: A detailed data set comprising 2367 data points was gathered, including ROP, MW, and SPP.
2. Preprocessing: The data set for this study was collected from an oil field in Iran, ensuring its high quality and reliability, with no incomplete or conflicting data. As a result, no further imputation or data-cleaning procedures were necessary.

- Model section:
  In this study, several advanced ML models were employed to predict ECD under standard conditions. These models encompass different forms of neural networks and regression techniques, which utilize various optimization algorithms to improve prediction accuracy in a range of scenarios.
- Model training and testing:

1. The data set was divided into training and testing subsets, with the former used for model training and the latter for final evaluation.
2. Performance metrics were determined to evaluate precision.

- Assessment and analysis:

1. A sensitivity analysis was conducted utilizing the relevance factor to assess the influence of each input parameter.
2. The Leverage technique was employed to establish the scope of applicability and identify outliers.

**Figure 1:**
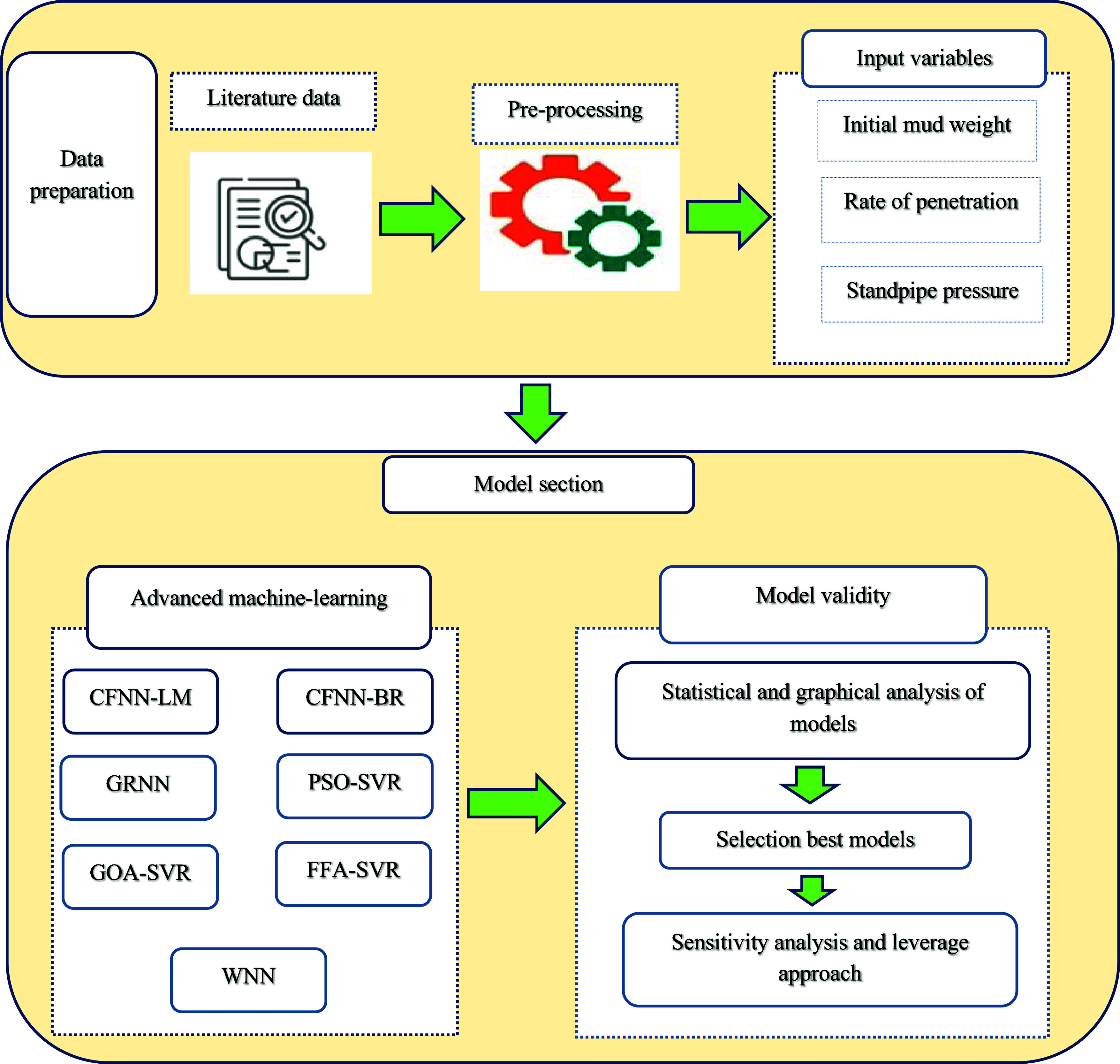
Prediction and validation process flowchart in this work.

The process of methodologies in this work is illustrated in Figure 1.

### 3.1 GRNN

**Figure 2:**
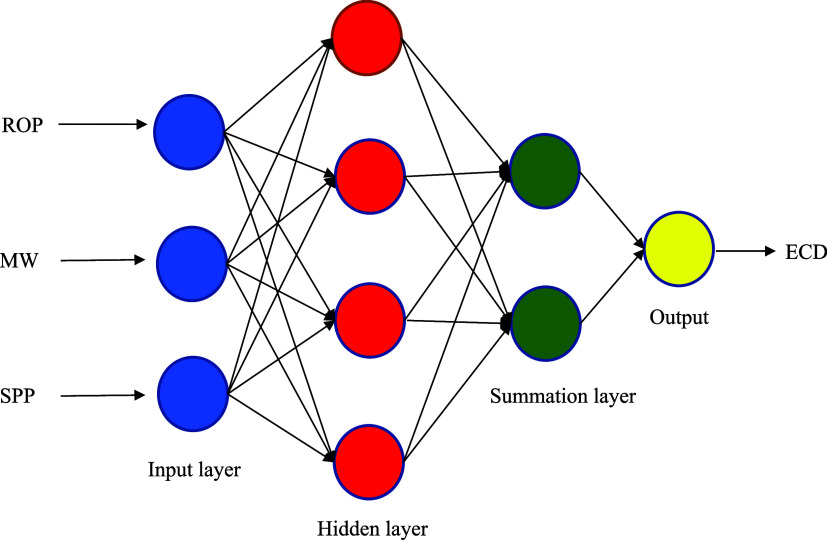
Diagram of the GRNN model applied in the present work.

The GRNN algorithm is a type of RBF that uses kernel regression networks. This model has four layers: input, pattern, summation, and output layers. It is based on nonlinear regression function estimation theory. In terms of structure, GRNN are comparable to RBF networks. The primary difference with GRNNs is that they utilize a dynamic linear function that incorporates two neurons in the output layer. One of these neurons processes the outputs from the pattern layer using specific weights, while the other neuron simply sums all outputs without applying any weights. The forecasted outputs for the input vector *X* by the GRNN are presented below.
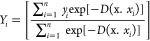
2
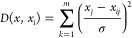
3In the above equation, *D* represents the Gaussian function, σ denotes the expansion factor, *X* refers to the input parameter, m signifies the count of input parameters, and *n* indicates the count of neurons in the model layer.^33^ A schematic of GRNN is depicted in Figure 2.

### 3.2 CFNN

**Figure 3:**
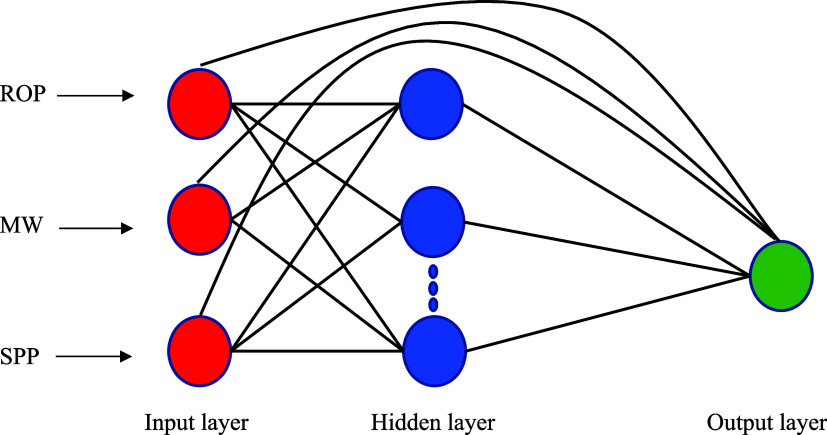
Typical CFNN model structure.

The CFNN architecture is made up of three layers: the input layer, the hidden layer, and the output layer. In the input layer, the number of inputs matches the number of nodes in the hidden layer, aiming to establish a connection between the input and output values.^34^ Neurons in the hidden layer are linked to neurons in other layers through a weight vector. To ascertain the number of neurons in the hidden or output layer, the vector derived from the input or hidden layer is initially multiplied by the weight vector. The activation functions modify the values of the neurons in the intermediate and output layers. In cascade networks, these functions typically encompass the inverse tangent and sigmoid functions. The benefit of this network, in contrast to multilayer perceptron networks, lies in its ability to account for both nonlinear and linear relationships between inputs and outputs.^35^ The cascade neural network has an additional weighted connection from the input layer to the output layers.^36,37^ A visual representation of the CFNN model is presented in Figure 3.

#### 3.2.1 CFNN-LM

In the CFNN network, the optimal weights and bias coefficients are determined by the LM algorithm using the nonlinear least-squares method.^38^ When using this algorithm, the determined local minimum response is not necessarily equal to the overall minimum response, even if there is a large distance from the final minimum response, the LM algorithm can achieve a suitable solution. Also, here it is not necessary to build a Hessian matrix and finally, the parameters of this matrix are calculated according to the provided formulas^39^
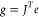
4
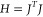
5Where *J*, *e*, and *T* are the Jacobin matrix, the direction of a network error, and a result of the Transpose matrix, respectively. To update the weights, using Newton’s equation, eq 6 is obtained
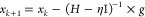
6Among the advantages of this training algorithm is the high speed of convergence to the response compared to other proposed neural network algorithms and the prevention of getting stuck in local minima, and the main disadvantage of the LM method is its need to store large matrices in memory, which itself requires a higher space.^40^

#### 3.2.2 CFNN-BR

In addition to the LM algorithm, the BR algorithm can also estimate weights and biases using the least-squares method.^41^ Therefore, by using an appropriate arrangement of weights and error squares, an acceptable structure is created.^42^ The weight of neurons is obtained using eq 7. This equation is as follows
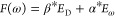
7where *a*, *B*, and *E*_D_ are total network errors, *E*_W_ is the sum of the squares of the weights in the network, and finally, *F*_W_ is the cost function. Weight distribution and network training are done by Gaussian distribution. It should be noted that the network weights are random variables in the BR algorithm. Using the Bayesian algorithm, defined parameters are selected to develop a space to reduce the cost function.^43^ Then, the calculations are transferred to the LM space and the local weights are updated. This process continues until the algorithm reaches the limit selected by the user.^44^

### 3.3 WNN

**Figure 4:**
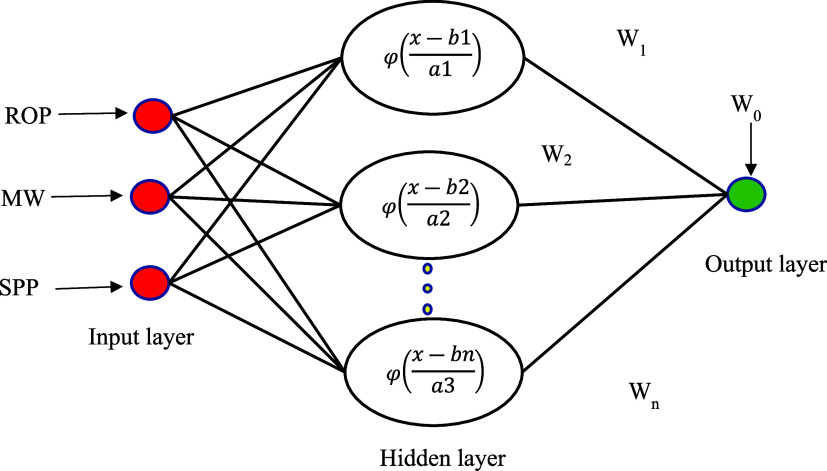
Schematic image of WNN model.

WNN is created from the combination of wavelet theory and neural networks. These networks have both the advantages and characteristics of neural networks and the attractiveness and flexibility of wavelet mathematical relationships and multiscale analysis.^45^ The wavelet algorithm is effective for function estimation, time series prediction, image processing, and signal noise detection. Wavelet networks, classified as layered feed-forward networks, consist of a single hidden layer that facilitates communication between the input and output layers.^46^ The output of WNN is measured using the following formula
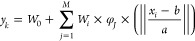
8Here, *M* represents the number of hidden nodes, *b* denotes the transfer factor, *a* refers to the spreading parameter, and φ*_J_* signifies the mother wavelet.^47^Figure 4 depicts a structure of WNN that is applied in this work.

### 3.4 SVR

Another ML algorithm that works on the basis of statistical learning theory is the SVR algorithm. In this algorithm, the effort is to establish a connection between the input data based on structural risk minimization.^17,48,49^ Structural risk minimization prevents local convergence common in neural networks. The need for fewer data and simple operation steps compared to neural networks are among the advantages of this method. In the SVR method, the input vectors are mapped in a multidimensional space.^50,51^ In this step, the kernel function is used to reduce the calculation steps in the high-dimensional space^52^
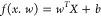
9The parameters of *W* and *b* are necessary to obtain the above equation. For this purpose, it must have a minimum value
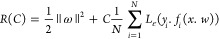
10In the above formula, coefficient *C* is a balancing parameter to determine the relationship between the size of the confidence margin ε and the amount of error in the training process. The function *Lε* is the Wepnick function, which is defined below
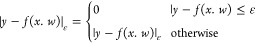
11The above problem can be rewritten as a solution of Lagrange’s Equations, by solving which the output function of the SVR model is expressed as the below equations^52^
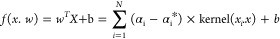
12
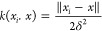
13The δ parameter controls the shape of the decision boundary and its optimal values can increase the accuracy of the model. In this research, to optimally select the three parameters *C*, ε, and δ in the presented regression model, meta-heuristic algorithms including PSO, GOA, and FFA were used, which are described below.^53^

### 3.5 PSO-SVR

**Figure 5:**
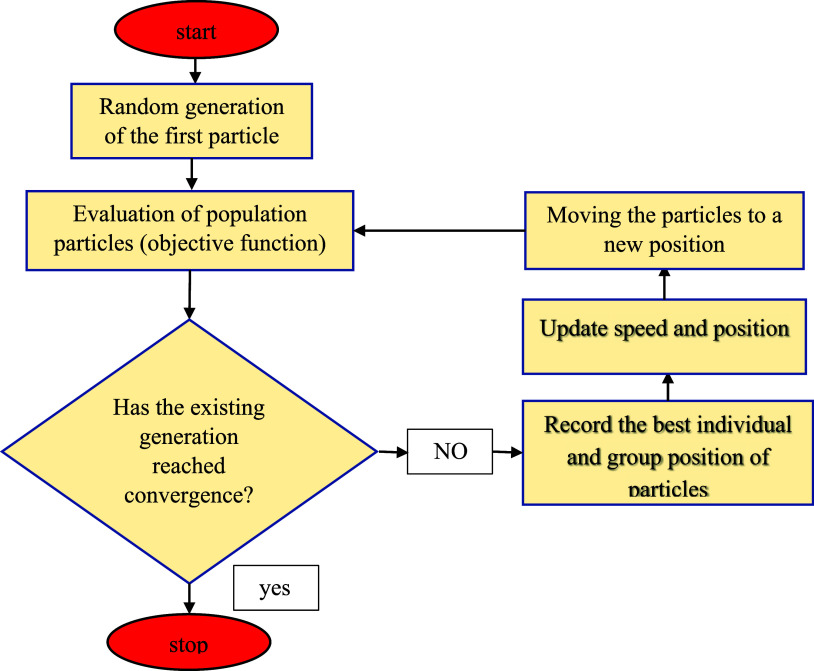
Schematic of PSO model utilized in this study.

The PSO-SVR draws inspiration from the collective flight of birds or the coordinated movement of fish groups. In each iteration, the new position of the particle can be established by utilizing the position velocity vector, with each member of the group characterized by its own velocity and position vectors. The algorithm was initially introduced to address continuous problems and later adapted to handle discrete problems, where it is referred to as binary PSO (BPSO).^54,55^ In each iteration, the particle’s position and velocity are updated using the following equations
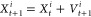
14
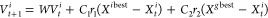
15where *X*_*t*_^*i*^, *X*_*t*+1_^*i*^, *V*_*t*_^*i*^, *X*^*i*best^, and *X*^*g*best^ are the current position of the particle, the next position of the particle in the probe space, the Current velocity of the particle, the velocity of the particle in the next position, the best individual position of the particle, and the best group position of the particles, respectively. Inertia coefficients W, C1, and C2 are individual and group learning coefficients of random numbers between zero and one to create the random position of particles in space. Figure 5 shows the PSO procedure.

### 3.6 FFA-SVR

**Figure 6:**
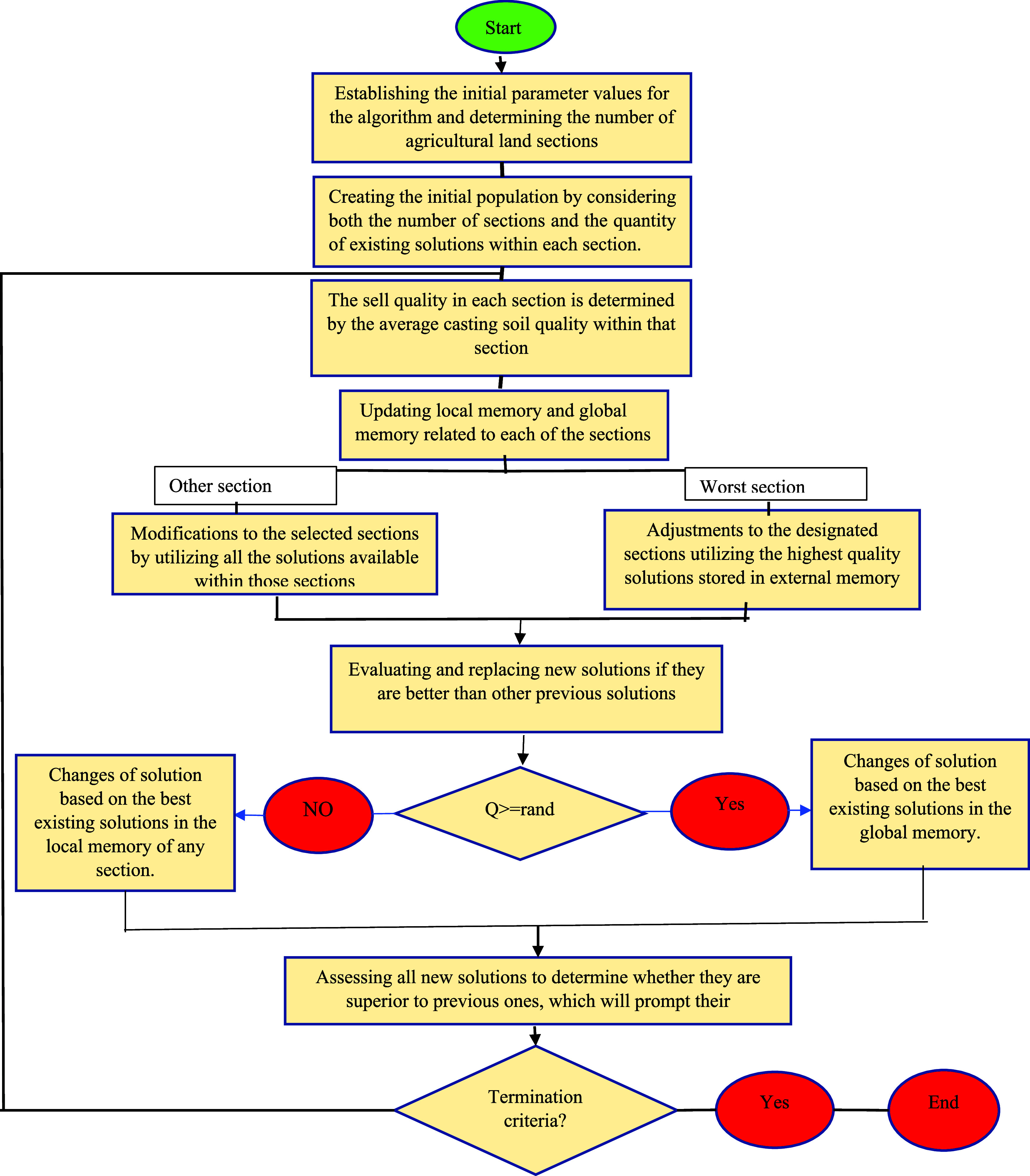
Framework of the FFA-SVR model employed in this work.

In the proposed algorithm, farmers assess the agricultural land and implement necessary modifications to the soil in each section, while also documenting the soil quality of each area. By using these notes, and the information they have about different parts of the agricultural land, they can make better decisions about improving the quality of the soil in each part of the agricultural land.^56^ After the soil quality of each part of the agricultural land has been determined, each part of the agricultural land that has the worst quality will be considered the most quantity and quality of special materials to change the quality of the soil of that part of the agricultural land. In this algorithm, the search space is segmented into multiple regions, with each region containing a nearby memory that records the most effective solutions identified within that specific area, as well as a global memory that retains the best solution found throughout the whole search space.^57^ The soil population initialization and search space are defined by the following formula
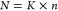
16
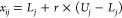
17In this context, *N* represents the total population size, *k* denotes the number of sections, and *L* and *U* indicate the lower and upper limits, respectively. Additionally, *x* and *r* are random numbers generated within the range of zero to one. *j*, *i*, and *n* represent the number of optimization variables, the total population, and the number of solutions available in each part of the agricultural land. In the next step, the suitability of the solutions in each part of the search space is determined and the local memory in each part as well as the global memory are synchronized.^58^ The flowchart of the FFA is shown in Figure 6.

### 3.7 GOA-SVR

The GOA was invented in 2017 by Saremi et al.^59^ The primary feature of grasshoppers in the childhood stage is slow movement and small steps. On the other hand, long-term and sudden movement is also a behavioral characteristic of adult grasshoppers.^60^ The search process is classified into exploitation and exploration by the GOR. During exploration, search agents are prompted to make abrupt movements, whereas, during exploitation, they tend to navigate locally. Optimization begins by creating a random set of solutions based on the equation below
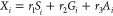
18where *X_i_*, *S_i_*, *G_i_*, and *A_i_* are the position of grasshopper *i*, social interaction between grasshoppers, and the force of gravity on the grasshopper *i* and wind flow, respectively. The coefficients of *r*_1_, *r*_2_, and *r*_3_ are the random numbers between 0 and 1 to show the random behavior of the grasshoppers. *S_i_* calculates with the below equation
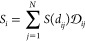
19where *d_ij_* is the distance between the *i*th and *j*th grasshopper in the group and to as

*d*_*ij*_ = |*x*_*j*_ – *x*_*i*_|, *d_ij_* is a unit vector between two grasshoppers and is expressed as . *S* function is to express the power of social force and it is defined as follows
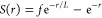
20where *f* denotes the attraction intensity and L signifies the length scale of attraction. The movement of the propellers is influenced by both repulsion and attraction among them, represented by SS. Variations in the values of *f* and *L* can lead to alterations in the outcomes produced by the algorithm. The force of gravity on each grasshopper and the effect of wind force on the propellers are expressed by the following equations
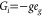
21
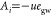
22where *gg* represents the gravitational constant and a unit vector directed toward the center of the Earth, *u* denotes the thrust constant, and *e*_gw_ is a unit vector indicating the direction of the wind. Finally, the search factors in this algorithm update themselves based on the below equations.
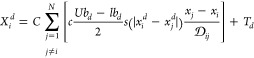
23where *Ub*_*d*_*and lb*_*d*_ are the upper and lower limits respectively at the *d*th distance, *T_d_* is the best solution discovered so far, and *C* is the reduction factor to shrink the comfort, repulsion, and attraction areas. Also, *N* is the count of grasshoppers. The first *C* in the above relationship balances the exploration and exploitation area around the objective function and increases the search around the objective function.^61,62^ The second C decreases the attraction, repulsion, or comfort zone, which can be calculated from the following formula
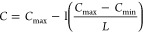
24where *C*_max_, *C*_min_, and *L* indicate the maximum, and minimum values, and number the number of repetitions, respectively.

### 3.8 GMDH

The GMDH algorithm is recognized as a self-organizing system. In 1972, Shankar conducted a study on this algorithm. Japanese and Polish scientists developed another variation of this algorithm.^63,64^ Their discovery revealed that GMDH is an effective method for pinpointing AI challenges, as well as for predicting both short and long-term future outcomes in random processes, while also discerning patterns within complex systems. Furthermore, regression analysis is recognized as a specific form of GMDH that uses the principles of statistical GMDH theory. In the GMDH algorithm, as a polynomial neural network (PNN) with layer structure, each layer of this network contains individual neurons. In this algorithm, each pair of neurons are independent of each other and are combined using a quadratic polynomial expression. Then, in the adjacent layers, new neurons are created, and finally, using the common independent parameters, a new structure is formed.^65^ The suggested correlation between input and output was presented using Volterra–Kolmogorov Gabor
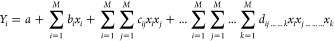
25*Y_i_* represents the output. Furthermore, *x_i_ x_j_*···*x_k_* is the input variable, and *a*, *b_i_*, *c_ij_*, *d*_*ij*···*k*_ display the coefficients of the polynomial. The variable *M* represents the count of independent parameters.

## 4 Evaluation of the Models

Statistical and graphic error analyses were combined to assess the reliability and accuracy of the proposed model using previously developed methods. Furthermore, applicability domain analysis and outlier detection methods were employed to assess the reliability of both the data points and the model.

### 4.1 Statistical Error Analysis

The validity of the models was investigated through measures including the determination coefficient (*R*^2^), average absolute percent relative error (AAPRE%), average percent relative error (APRE%), standard deviation (SD), and root-mean-square error (RMSE). Below are the statistical parameters provided^66^
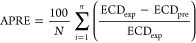
26
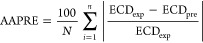
27
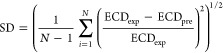
28
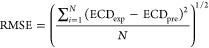
29
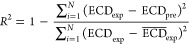
30where ECD_exp_, ECD_pre_, and  indicate the measured value of equivalent circulating density, predicted values of the desired parameter, and the mean of experimental data.

### 4.2 Graphical Error Assessment

1. Cross plot: This tool generates a scatter plot that displays the model estimates about the experimental data, along with a line of unit slope connecting the estimated and experimental values. This line serves to illustrate the ideal predicted outcome. The accuracy of proposed models increases with the accumulation of recorded data near the unit slope line.
2. Error distribution: In this method, the AAPRE is graphed against the independent variables to illustrate the distribution of errors around the zero-error line.
3. Cumulative relative frequency plot: This plot illustrates the proportion of data points predicted by the model within a given range of relative error.

### 4.3 Model Applicability and Outlier Detection

Data points that markedly differ from the majority of the data set are called outliers. These outliers signify uncertainty and can negatively impact the predictive accuracy of the developed method. To address this issue, statistical tests have been created to identify these outliers. In this study, we used the leverage technique to identify outliers and assess the scope of applicability of the developed models.^66^

## 5 Results and Discussion

In this work, seven ML techniques, including CFNN-LM, CFNN-BR, GRNN, WNN, PSO-SVR, FFA-SVR, and GOA-SVR were employed to estimate ECD. Furthermore, the GMDH method was used to build a new empirical correlation. SPP, ROP, and MW were identified as the model input variables. A comprehensive data set consisting of 2367 real-field data points was randomly partitioned into two subsets for model development, as detailed below:

(a) Training Set: This subset was utilized for model development, optimization, and performance improvement. During the training process, the model develops a relationship between the input parameters and the output by progressively modifying biases and weights to minimize the discrepancy between the observed and predicted results.
(b) Test set: This subset was considered to assess the model’s predictive performance and evaluate its accuracy.

In this study, 70% of the data set was allocated for training the models, while the remaining 30% was reserved for evaluation during the testing phase. Fine-tuning the hyperparameters is essential for reducing prediction errors in ML models.^67^ The hyperparameters of each AI model were carefully tuned to optimize performance and prevent overfitting. Techniques such as grid search and trial-and-error were employed to identify the best configurations for each algorithm. The principal hyperparameter values obtained during the modeling process are presented in Table 3.

**Table 3:** Hyperparameters and Features of All Models

model	parameter	value
CFNN-BR	hidden layer Size	[8 6]
	transfer function	tansig - logsig - purelin
CFNN-LM	hidden layer Size	[8 6]
	transfer function	tansig - logsig - purelin
WNN	hidden layer neurons	8
	B-spline wavelet activation function	Fb = 0.3, *m* = 1.5, and Fc = 0.25
GRNN	spread	0.022
PSO-SVR	number of swarms	30
	inertia weight	0.8
	*C*_1_ = *C*_2_	2
	iterations	200
FFA-SVR	farmland sections	4
	solutions per section	50
	population size	200
	α	0.6
	β	0.4
	w	1
	Q	0.5
	iterations	200
GOA-SVR	population size	30
	*C*	0.01
	ε	0.02
	γ	0.1
	iterations	200
	objective function	MSE
GMDH	neuron input	2
	initial layer width	2
	max number of layer	12
	error function	MSE

It is essential to recognize that strong performance during the training phase does not necessarily guarantee the model’s reliability. Therefore, assessing the models with the reserved data set is fundamental to verifying their accuracy and robustness. Next, a comprehensive evaluation of the developed models was performed using both statistical and graphical error analysis techniques. In addition, a sensitivity assessment was carried out to evaluate how input parameters influence the outputs of the models.

As a preliminary step, a correlation was formulated using the input parameters to predict the ECD.

### 5.1 Correlations’ Development

The mathematical equation was provided by the GMDH algorithm to estimate the ECD based on the parameters of MW_S_, ROP, and SPP. The obtained GMDH model is as follows
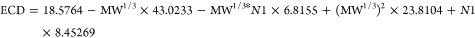

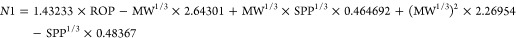
In this equation, MW represents surface mud density, SPP is stand pipe pressure, and ROP is the rate of penetration.

### 5.2 Statistical Assessment of the Models

As stated earlier, *R*^2^, APRE, AAPRE, SD, and RMSE were employed to evaluate the accuracy of the model’s predictions. The results of the statistical error metrics derived from the training, testing, and overall data sets for all models are presented in Table 4. Based on statistical principles, a model is considered more accurate and reliable when its *R*^2^ value is closer to 1, and the RMSE, AAPRE, APRE, and SD values in the modeling process are lower. Based on the statistical results, the GOA-SVR model achieved the most accurate ECD predictions, with the lowest AAPRE values of 0.0869% for the total data set, 0.0823% for the training set, and 0.0975% for the testing set. Following this, the PSO-SVR model ranked second in accuracy, with AAPRE values of 0.0897% (total data), 0.0827% (training), and 0.1060% (testing). The FFA-SVR model secured the third rank among the proposed models, achieving a total AAPRE of 0.0997%, with values of 0.0928% for the training data set and 0.1160% for the testing data set.

**Table 4:** Statistical Metrics Analysis for the Suggested Models and Correlation

model		APRE %	AAPRE %	RMSE	SD	*R*^2^
	train	–0.0013	0.1749	0.0031	0.0025	0.9993
CFNN-BR	test	–0.0039	0.1803	0.0033	0.0027	0.9992
	total	–0.0200	0.1765	0.0032	0.0026	0.9993
	train	–0.0049	0.1240	0.0025	0.0021	0.9996
CFNN-LM	test	–0.0018	0.1297	0.0026	0.0022	0.9994
	total	–0.0006	0.1257	0.0025	0.0021	0.9996
	Train	–0.0409	0.5604	0.0094	0.0078	0.9927
GRNN	test	–0.0670	0.5633	0.0098	0.0082	0.9917
	total	–0.0488	0.5613	0.0095	0.0079	0.9938
	train	–0.0006	0.1605	0.0029	0.0024	0.9993
WNN	test	0.0075	0.1635	0.0058	0.0042	0.9972
	total	0.0018	0.1614	0.0040	0.0030	0.9989
	train	0.0053	0.0827	0.0023	0.0011	0.9996
PSO-SVR	test	–0.0122	0.1060	0.0037	0.0033	0.9989
	total	0.0004	0.0897	0.0028	0.0024	0.9995
	train	0.0012	0.0928	0.0022	0.0019	0.9996
FFA-SVR	test	–0.0080	0.1160	0.0036	0.0027	0.9989
	total	–0.0158	0.0997	0.0027	0.0021	0.9995
	train	0.0046	0.0823	0.0024	0.0020	0.9995
GOA-SVR	test	0.0087	0.0975	0.0045	0.0037	0.9983
	total	0.0058	0.0869	0.0032	0.0026	0.9993
	train	–0.0275	0.1930	0.0036	0.0031	0.9910
GMDH	test	0.1815	0.1892	0.0026	0.0022	0.9818
	total	–0.0007	0.1859	0.0032	0.0027	0.9991

Furthermore, Table 5 is presented to compare the performance of GOA-SVR and GMDH models with previous models statistically. The results presented in this table suggest that the GOA-SVR and GMDH methods demonstrate a lower AAPRE compared to the previous models. Specifically, the AAPRE values for these algorithms are calculated to be 0.0869% and 0.1859%, respectively.

**Table 5:** Comparing the Statistical Evaluation of the GOA-SVR and GMDH Models with Previous Models for Estimating ECD

ECD model	data point	AAPRE %	RMSE	*R*^2^
Abdelgawad et al.^23^	2367	0.22	0.00562	0.98
Gamal et al.^32^	3570	0.19	*	0.99
Alsaihati et al.^19^	1152	*	0.35	0.95
GOA-SVR	2367	0.0869	0.0032	0.9993
GMDH	2367	0.1859	0.0032	0.9991

### 5.3 Graphical Evaluation of the Models

**Figure 7:**
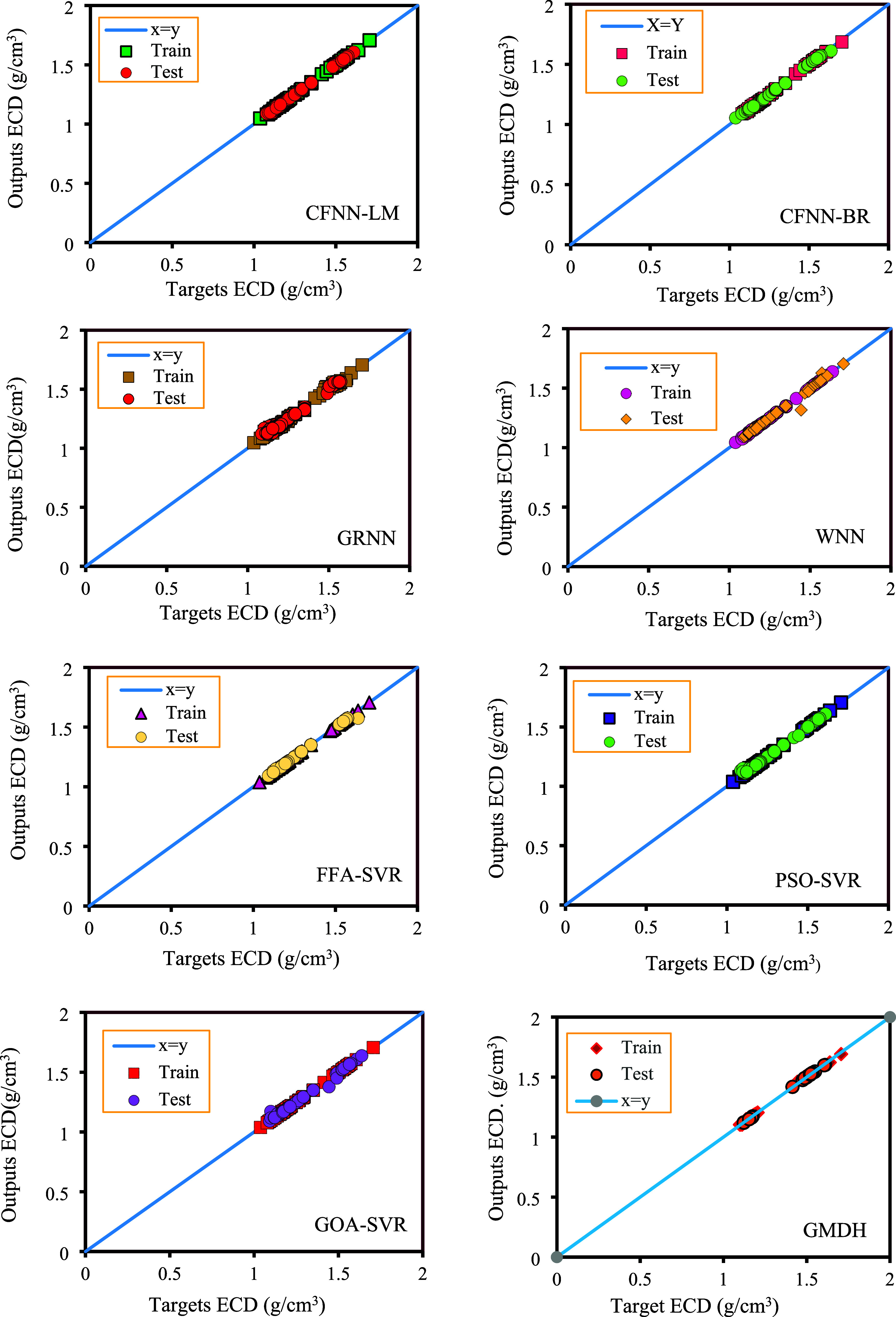
Cross-plots of the suggested models for predicting ECD.

In this section, graphical error analysis is used to illustrate the reliability and precision of the models. Therefore, three types of graphical analyses, including cross-plots, relative error distribution, and cumulative frequency diagrams, were thoroughly analyzed. Initially, the cross plots of all models are presented in Figure 7. As previously mentioned, the closer the data points are to the *X* = *Y* line, the higher the model’s accuracy in predicting the ECD. As shown in Figure 7, the GOA-SVR models exhibit the closest data points to the *X* = *Y* line in comparison to the other proposed models and correlations, demonstrating their significant robustness and validity for predicting the ECD. In Figure 7, there is a clear and strong correspondence between the real data and the values anticipated by the models.

**Figure 8:**
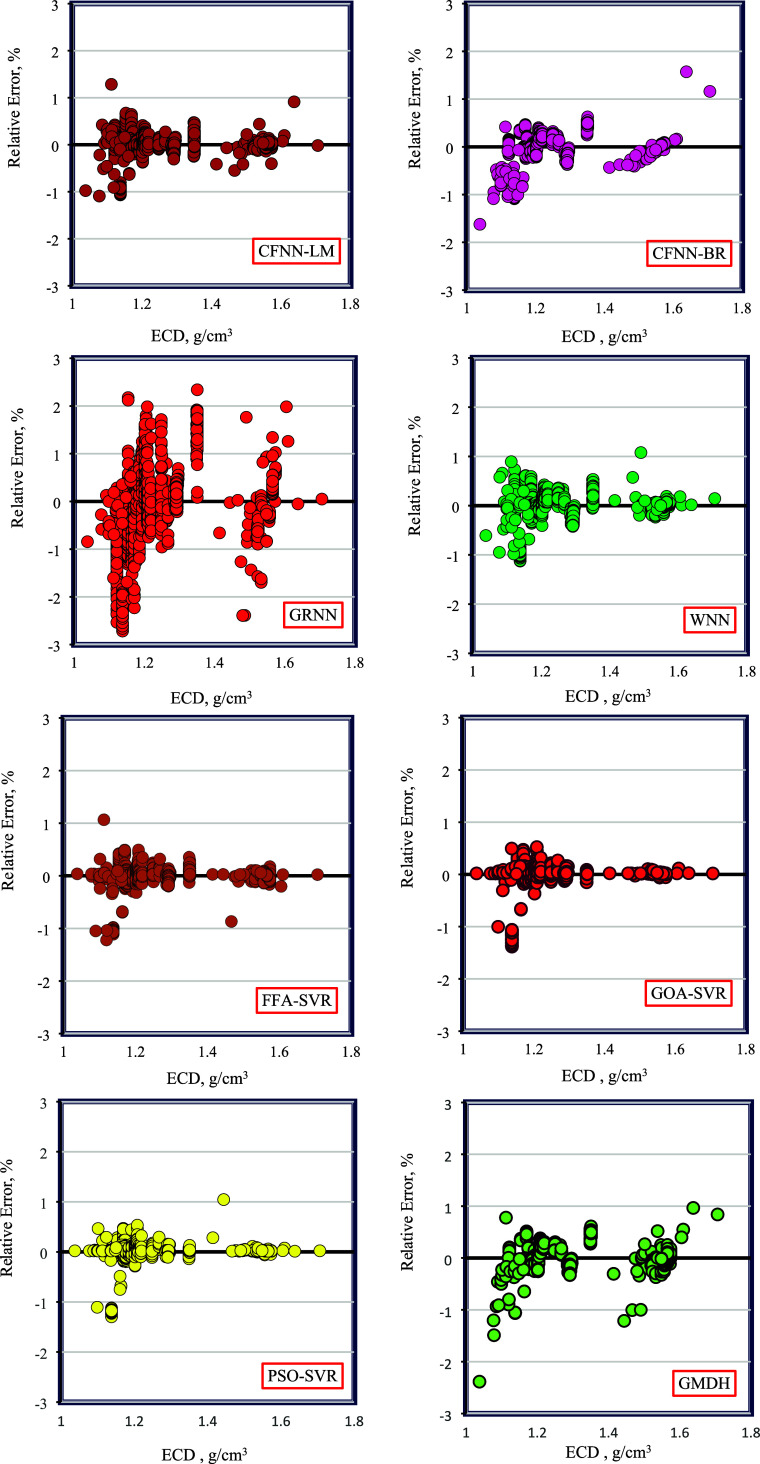
Relative error distribution plots for the models.

Subsequently, Figure 8 displays the distribution of relative error to ECD for the models created in this work. In the error distribution plot, a broader spread of data around the zero-error line typically reflects lower model accuracy, while a tighter concentration of points near this line indicates higher model accuracy. The data for the GOA-SVR method clusters more closely around the zero-error line compared to others, suggesting minimal deviation and demonstrating high accuracy for this model. Based on this analysis, it can be inferred that the GOA-SVR model is the most precise model for estimating ECD.

**Figure 9:**
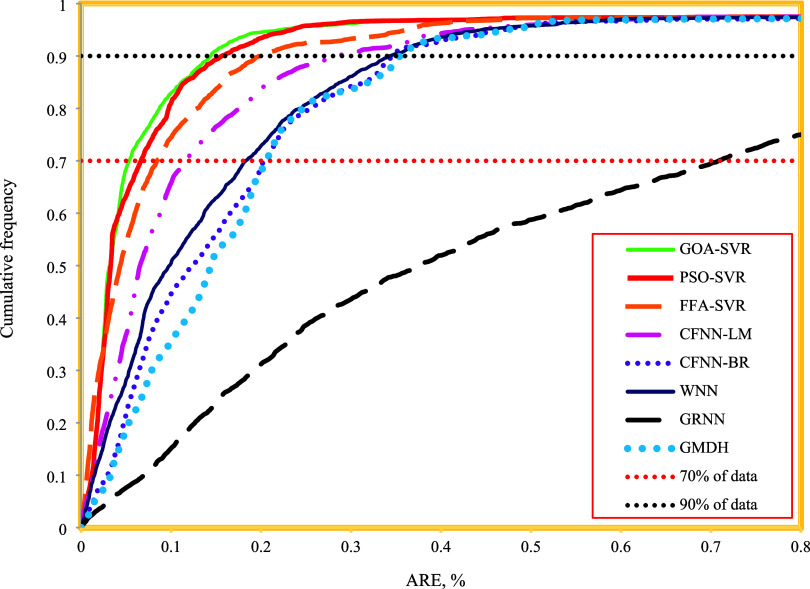
Cumulative frequency plot versus ARE for the suggested models.

While the models presented in this research demonstrate high accuracy, it is crucial to introduce the model with the highest precision. To achieve this, Figure 9 illustrates a cumulative plot of the developed models, providing a visual representation that facilitates a comparative analysis of their performance. Considering the red horizontal dashed line representing 70% of the data in the figure, it can be observed that the GOA-SVR, PSO-SVR, FFA-SVR, CFNN-LM, WNN, CFNN-BR, GMDH, and GRNN models exhibit absolute relative errors of 0.056%, 0.069%, 0.088%, 0.12%, 0.2%, 0.22%, 0.23%, and 0.71%, respectively. Similarly, approximately 90% of the values estimated by the GOA-SVR model exhibited an ARE of less than 0.15%, whereas the error values for the other models exceeded this threshold. These findings, supported by additional statistical and graphical analyses, demonstrate that the GOA-SVR model achieves high precision in forecasting ECD.

**Figure 10:**
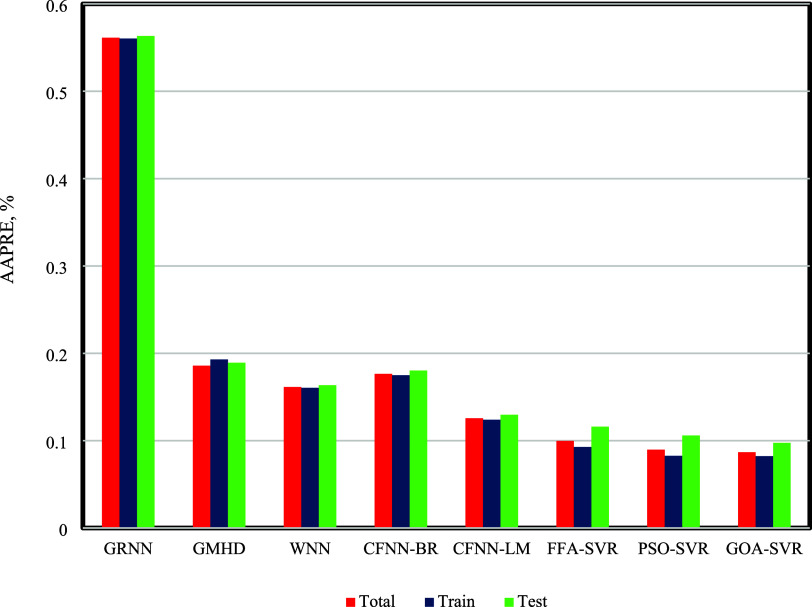
Accuracy of the suggested models based on AAPRE.

Next, a comprehensive comparison based on AAPRE was conducted to evaluate the credibility of the presented models in this investigation. The outcomes of this comparison are presented in Figure 10 for the overall data, training data, and test data for these models. The accuracy of the proposed model based on AAPRE is shown in Figure 10. As illustrated in the figure, the GOA-SVR model exhibits the optimal performance, with an AAPRE of 0.0869% for the overall data set, 0.0823% for the training data set, and 0.975% for the test data set. Additionally, the ranking of the models based on their performance, can be presented as follows:

### 5.4 Sensitivity Analysis

Sensitivity analysis is mainly utilized to evaluate the influence of different input variables on the model’s outputs. This method employs the relevancy factor (*r*), which is explained as follows^68^
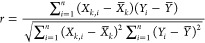
31where *X*_*k.i*_ and *X̅*_*k*_ represent the ith value and the mean value of input *k*, respectively. Furthermore, *Y*_*i*_ and *Y̅* represent the ith value and the mean value of the model’s output, respectively. This method assigns values between −1 and 1 to input parameters, where higher absolute values indicate stronger influence on the output, and zero indicates no correlation. Positive and negative coefficient indicate a direct and inverse effect on the output, respectively.^69^

**Figure 11:**
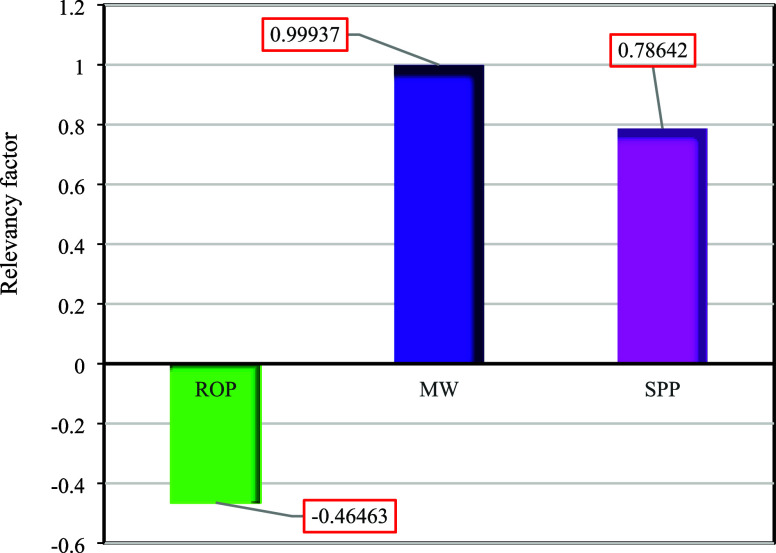
Relative influence of the model inputs on ECD.

Figure 11 illustrates input parameters’ relative influence and significance on ECD. As observed, MW exhibits the greatest influence in the proposed model, with a significance of approximately 0.99. Following MW, SPP demonstrates the next highest impact, with an importance of around 0.78. Both parameters exert a positive effect, whereas the ROP parameter, with a value of −0.46, has a negative impact on the model’s output.

### 5.5 Leverage Technique

**Figure 12:**
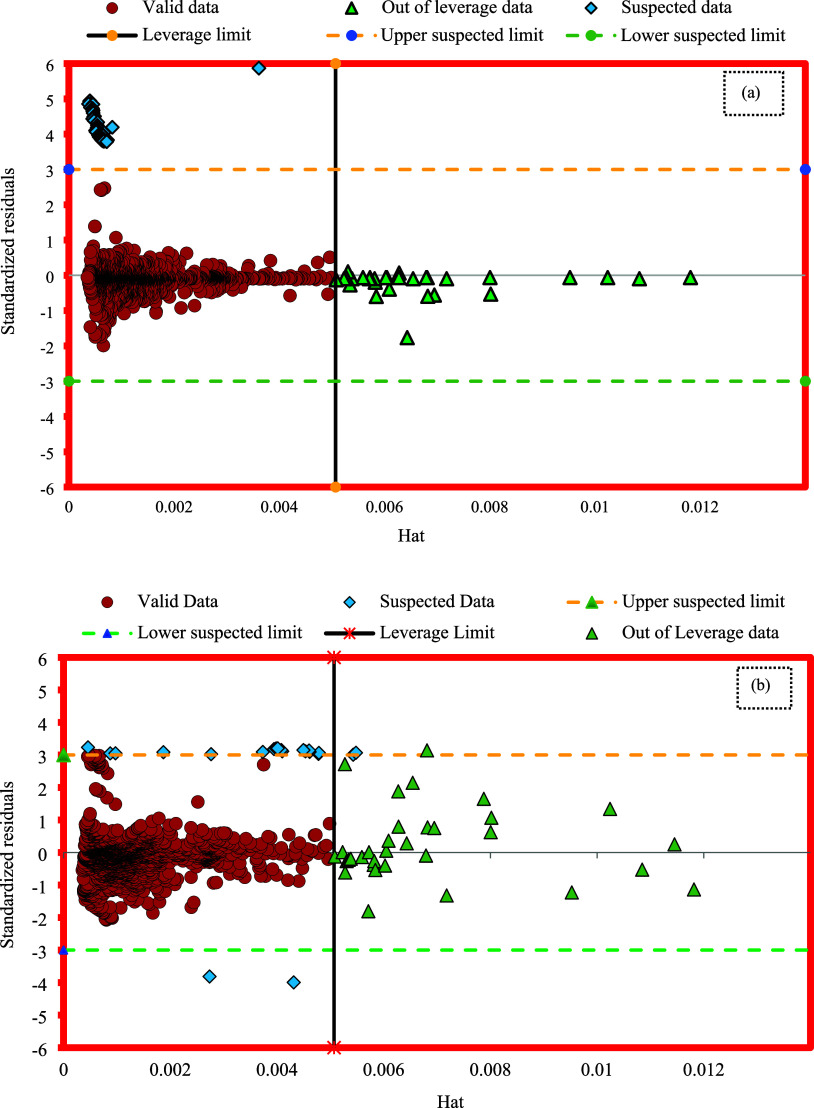
William’s plots for the suggested models: (a) GOA-SVR, (b) GMDH.

In this study, the leverage technique^70−72^ was used to assess the validity range of the proposed GOA-SVR model and GMDH correlation, enabling the identification of any potentially unreliable data. In this method, the discrepancies between the model’s predictions and the experimental data are referred to as standardized residuals (SR).^66^ Considering *h*_*i*_ as the ith leverage value and MSE as the mean squared error, the SR values are presented below
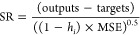
32Standardized residuals are embedded within the Hat matrix. Additionally, hat indices correspond to the elements along the main diagonal of the Hat matrix. Let *T* denote the transpose of matrix *X*, where *X* is a (*k* × *L*) matrix comprising *k* rows (data points) and *L* columns (input variables). The hat indices are derived from the Hat matrix, which is formulated as follows
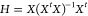
33Furthermore, the critical leverage (H*) remains constant for a given data set and is determined as follows
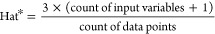
34The Williams plot is typically used to visually show the operational scope of the model and to identify questionable data within the data set, as shown in Figure 12 for the GOA-SVR and GMDH models employed in this study. In this context, high-leverage and undesirable points refer to those with SR values greater than 3 and less than −3, regardless of their Hat values.^66,68^ As illustrated in Figure 12, 59 data points (∼2.5%) for the GOA-SVR model and 29 data points (∼1.3%) for the GMDH model were identified as suspicious, indicating they were considered laboratory-suspected. Furthermore, data points with SR values between −3 and 3 and a Hat value exceeding H* (0.00507) are categorized as good high leverage. As depicted in Williams’s plot, 30 data points (approximately 1.3%) for the GOA-SVR model and 42 data points (approximately 1.8%) for the GMDH model were identified as potential outliers. This suggests that, despite accurate estimations, these data points fall outside the model’s applicability domain and deviate from the majority of the data set. The results obtained from the leverage approach validate the precision of the ECD database and highlight the strong reliability of both GOA-SVR and GMDH models in accurately anticipating the ECD.

## 6 Conclusions

In oil well drilling, the ECD is essential for maintaining wellbore stability and preventing blowouts. ECD combines drilling fluid pressure with hydrostatic pressure to counterbalance formation pressures, enabling safer drilling operations. The novelty of this work lies in the use of fewer inputs for modeling, which enhances the simplicity and efficiency of the approach. Furthermore, several advanced ML models, including CFNN-BR, CFNN-LM, WNN, GRNN, GOA-SVR, PSO-SVR, and FFA-SVR, have been utilized to predict ECD. The models utilized in this study demonstrated a high degree of adaptability to complex data and varying conditions, resulting in superior performance in predicting ECD compared to other models. Additionally, these models exhibited robustness to noisy and inconsistent data, enabling reliable predictions even in the presence of discontinuous and irregular data sets. Moreover, the empirical relationship developed in this work outperforms existing models in terms of accuracy, providing a more reliable and trustworthy predictive framework for ECD estimation. The models were trained and evaluated using three input parameters including MW, ROP, and SPP on a data set consisting of 2367 data points, and their performance was evaluated through statistical analysis and graphical representations. The key outcomes of this study can be outlined as follows:

1. Among the suggested models, the GOA-SVR model indicated exceptional accuracy, achieving an overall AAPRE of 0.0869%. The PSO-SVR and FFA-SVR models, following the GOA-SVR model, showed the next highest levels of accuracy, with AAPRE values of 0.0897 and 0.0997%, respectively. These findings underscore the superior performance of the GOA-SVR model in predicting ECD.
2. The outcomes of the correlation developed using the GMDH method indicated that this approach achieves the highest level of accuracy compared to the previously established models.
3. The sensitivity analysis indicated that MW has the strongest influence on the model (0.99), with SPP following at 0.78, both positively affecting the output. On the other hand, ROP shows a negative effect with a value of – 0.46.
4. The outcomes of the leverage method validate the precision of the ECD data set and highlight the strong reliability of the GOA-SVR and GMDH models in ECD estimation. Additionally, 2.5 and 1.3% of the data were identified as suspect data in the GOA-SVR and GMDH models, respectively, while 1.3 and 1.8% were classified as potential outliers.

Finally, it is recommended that future research extend the models by incorporating diverse field data and applying them to specific drilling conditions, such as fractured formations and HPHT. Additionally, integrating physical modeling approaches with data-driven methods could substantially improve the accuracy and generalizability of ECD predictions.

## Nomenclature

ML: machine learning
APRE: average percent relative error
ECD: equivalent circulating density
ROP: rate of penetration
SPP: standpipe pressure
MWs: surface mud weight
AAPRE: average absolute percent relative error
CFNN-LM: cascade forward neural network with Levenberg-Marquardt
WNN: wavelet neural network
FFA-SVR: support vector regression combined with farmland fertility algorithm
GOA-SVR: support vector regression combined with a grasshopper optimization algorithm
GMDH: group method of data handling
*R*^2^: root mean square error
AI: artificial intelligence
GRNN: general regression neural network
SD: standard deviation
CFNN-BR: cascade forward neural network with Bayesian network
